# A Conversational Platform (Okaya) for Multimodal Digital Biomarkers of Fatigue, Cognition, and Mental Health: Feasibility Observational Study

**DOI:** 10.2196/87054

**Published:** 2026-04-01

**Authors:** Matthew So, Michael Sobolev, Gregory Menvielle

**Affiliations:** 1 Okaya, 540 Mohawk Drive, Boulder, CO, 80303, United States, 1 9259841028; 2 Cornell Tech, New York, NY, United States

**Keywords:** digital biomarkers, multimodal sensing, computer vision, speech acoustics, natural language processing, artificial intelligence, AI

## Abstract

**Background:**

Collection of multimodal data (video, audio, and text) can yield digital biomarkers relevant to mental health, fatigue, and cognition. However, the feasibility and signal characteristics in operational populations remain underexplored.

**Objective:**

The objectives of this study were to (1) extract an evidence-based library of vision, speech, and language features; (2) assess the feasibility of a fully remote conversational platform (Okaya) for collecting analyzable multimodal data; and (3) conduct preliminary signal checks for depression, fatigue, and cognition.

**Methods:**

Participants were recruited from the US Air Force and US Space Force. All participants completed the Okaya check-in, which included a voice conversation with a large language model. A total of 66 visual, acoustic, and text features were extracted from each interaction between the participant and the large language model. For validation purposes, the study also collected measures of depression (Patient Health Questionnaire–9), fatigue (Cancer Fatigue Scale), and cognition (trail making test). We evaluated the feasibility of the platform and correlation between the extracted features and the validated assessments.

**Results:**

A total of 8 unique participants contributed with 62 sessions over a period from March 6, 2025, to August 6, 2025. The platform was deemed feasible as 6 of the 8 participants opted to complete more than one session, and the 3 participants who provided feedback reported high overall experience and usability. From the data perspective, preliminary correlations produced significant results for multiple potential digital biomarkers, including (1) pitch (*P*=.047), volume SD (*P*=.04), volume slope (*P*=.04), automated readability index complexity (*P*=.047), Flesch-Kincaid complexity (*P*=.04), and Gunning Fog complexity (*P*=.04) for depression; (2) pitch (*P*=.009), volume SD (*P*=.007), volume slope (*P*=.02), average F2 formant frequency (*P*=.03), Gunning Fog complexity (*P*=.049), and eyelid droop (*P*=.047) for fatigue; and (3) shimmer (*P*=.03) for cognition. We also observed how features varied over time among participants with multiple sessions.

**Conclusions:**

The conversational and artificial intelligence–enabled platform was feasible among an operational sample as a method to collect multimodal data correlated with depression, fatigue, and cognition. These results align with those for previously discovered digital biomarkers of mental health, fatigue, and cognition and inform the development of personalized models for each user while detecting anomalies in a remote monitoring setting.

## Introduction

Mental health, fatigue, and cognitive functioning are critical to overall well-being and operational performance, yet their assessment often relies on intermittent self-report or clinician-administered instruments. Such methods, while clinically validated, can be burdensome, subjective, and limited in their ability to capture dynamic changes over time [1-9]. Mobile health technology provides an alternative solution due to the ability of apps and sensors to collect high-fidelity and high-frequency data pertaining to activity, behavior, symptoms, and cognition [10-12]. With increasing demands for continuous and remote monitoring—especially in occupational and high-performance settings—digital biomarkers have emerged as a promising avenue for objective and scalable assessment of psychological and cognitive states [13-16].

Recent advances in computer vision, speech acoustics, and natural language processing have enabled the extraction of behavioral and physiological signals from everyday digital interactions. Multimodal sensing that integrates video, audio, and language data provides an opportunity to quantify indicators of affect, fatigue, and cognitive load with greater ecological validity than traditional laboratory tests [11,17]. Prior research has demonstrated associations between acoustic features (eg, pitch variation [18-23], jitter [18,19,24-26], and spectral harmonicity [25-28]), facial expression dynamics (eg, eye gaze [29-32] and microexpressions [33]), and linguistic markers (eg, sentiment [28,34,35], complexity [28,36], and emotional valence [28,35,37-41]) with symptoms of depression, stress, and cognitive decline. However, most existing work has been conducted in tightly controlled environments, and less is known about the feasibility of collecting such data in naturalistic or operational contexts [42].

Conversational platforms, especially those powered by large language models, introduce new opportunities for naturalistic data capture [43-46]. By engaging users through dialogue, these systems can simultaneously elicit and record multimodal signals—speech, facial behavior, and language—while maintaining a familiar and low-burden interaction format [47-52]. Such interfaces align with the increasing integration of conversational artificial intelligence (AI) into health, wellness, and performance domains, offering a pathway toward longitudinal, user-centered monitoring of mental and cognitive health. Nevertheless, empirical evaluation of these systems’ feasibility and usability remains limited, particularly in real-world populations such as operational and occupational groups [53,54]. These groups can also be more susceptible to experiencing mental and cognitive health challenges due to their job functions [55,56].

The objective of this study was to evaluate the feasibility of a fully remote conversational platform, Okaya, for collecting analyzable multimodal data (video, audio, and text) in an operational population from the US Air Force (USAF) and Space Force. Specifically, we sought to determine whether participants would engage with the platform across multiple sessions and whether the system could reliably extract a comprehensive set of visual, acoustic, and linguistic features suitable for future digital biomarker modeling of depression, fatigue, and cognition. Finally, we present a case study for the design of a risk score based on a model of extracted digital biomarkers to illustrate the future potential of the platform.

## Methods

### Study Population

This study was part of an existing Small Business Innovation Research Direct to Phase II contract with the USAF, and a pool of potential participants from the 71st Special Operations Squadron, provided by the USAF, was introduced to the Okaya platform as part of a pilot program. Participation was entirely optional. A total of 8 unique users participated in the study, contributing 62 sessions (median 2.5, range 1-38, IQR 1.5-7.5). Participants provided data during the period from March 6, 2025, to August 6, 2025.

### Ethical Considerations

Ethics approval for this study was obtained from Sterling Institutional Review Board (approval 12300-GMenvielle), and all participants signed an informed consent statement that specified anonymized access to research records, potential risks, and voluntary participation. No data were collected except for email addresses, which were required to access the Okaya platform. No demographic or sample composition information was collected due to requirements from the USAF. Participants received no compensation for taking part.

### Procedure and Data Collection

#### Overview

**Figure 1.**
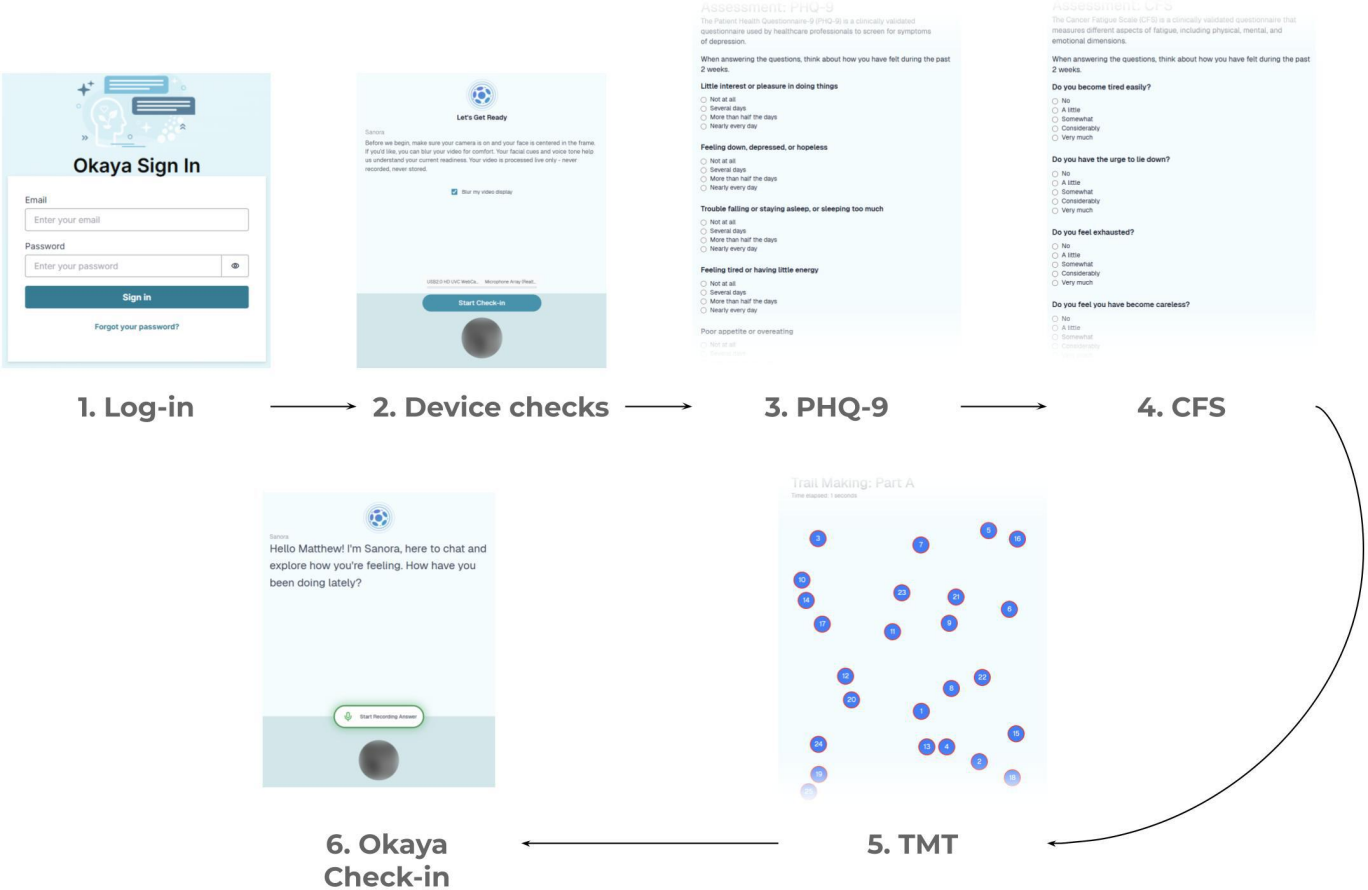
Illustration of the Okaya platform and data collection. The 6 steps for a successful sample collection include log-in, device checks, Patient Health Questionnaire–9 (PHQ-9), Cancer Fatigue Scale (CFS), trail making test (TMT), and Okaya check-in.

Completion of Okaya check-ins [57] was entirely voluntary. Data collection took place on the Okaya website, and users were allowed to select the device they preferred to access the website with, such as smartphones, tablets, or laptops. The browser used to access the website was also free to select by the user. Once users had successfully acknowledged the consent statement and registered for an Okaya account, they could complete a workflow as described in Figure 1. Participants were asked to complete 3 assessments: the Patient Health Questionnaire–9 (PHQ-9) [58], Cancer Fatigue Scale (CFS) [59], and trail making test (TMT) [60].

**Figure 2.**
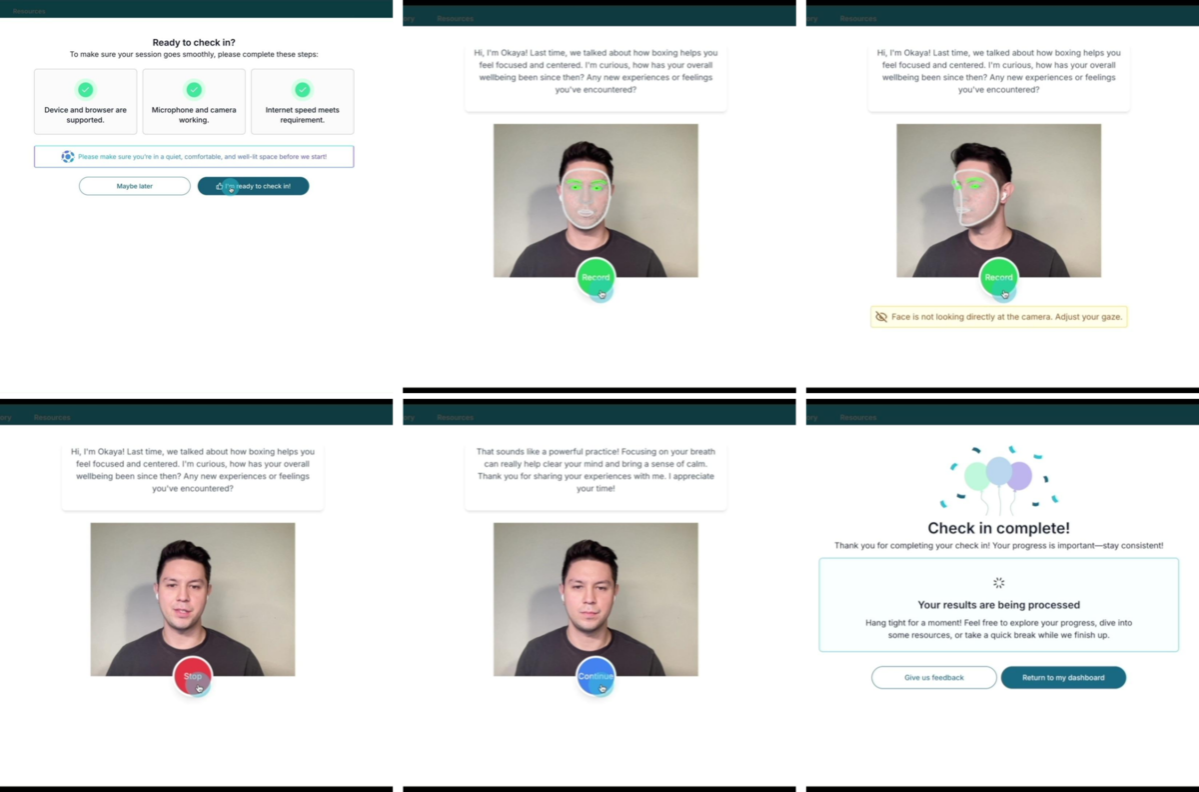
Screenshots of the Okaya check-in. Permission was obtained from the user depicted.

Upon successful completion of all 3 assessments, users were presented with a text and voice prompt from a large language model (using OpenAI’s GPT-4o-mini model with a custom system prompt, referred to as “Sanora”), shown in Figure 2. Users were first required to meet the device and browser requirements, grant permission to access the microphone and camera, and meet an upload speed requirement of 2 Mb/s. Video was recorded continuously, and users could choose between webcam video, blurred video, a landmark representation of their face, an outline of their face, or a set of axes denoting the location of their face, designed to accommodate users with differing levels of comfort with being recorded. Although video was shown on the interface, raw video was not sent to our servers; instead, a set of facial landmarks was extracted via Google MediaPipe. These landmarks were automatically extracted using a proprietary machine learning model, and 478 landmarks were output, each with an x, y, and z coordinate, as well as 52 “blendshapes,” each representing various facial expressions. The check-in interface, as shown in Figure 2, included the text of Sanora’s prompt at the top; the representation of the user’s webcam video at the center; and a control button, either “Record,” “Stop,” or “Continue,” at the bottom of the screen.

After passing device checks and clicking “I’m ready to check-in,” Sanora would generate a prompt for the user to respond to asking about their general mental well-being, emotions, or experiences they had had recently. Upon clicking “Record” to record a response to Sanora, the user’s microphone started recording, and an automatic transcription service from Amazon Transcribe started transcribing their speech until the user clicked “Stop” and stopped recording. Both the audio recording and transcribed text were sent back to the Okaya servers. Sanora then used the transcribed text to generate a response. This process continued for 4 turns (5 messages from Sanora and 4 responses from the participant). Upon conversation completion, the user clicked “Continue” to submit the session for analysis, after which a completion screen would indicate that the results were being processed. They would then be redirected to the research dashboard.

All back-end processing took place on Amazon Web Services infrastructure, with communication between the website (front end) and back end occurring via Representational State Transfer application programming interface calls. Data were also stored on Amazon Web Services infrastructure.

#### Feature Extraction

From the landmarks, audio, and transcribed text, we extracted 66 features based on the literature. A full list and descriptions of each implemented feature can be found in Multimedia Appendix 1, whereas summaries are shown in Tables 1-3.

**Table 1.** Mapping features for depression.

Feature	Theoretical reasoning	References	Implemented features
Visual features			
Flat affect	A predominant lack of facial expressiveness can be observed in some individuals with depression. This might manifest as a “blank” or “emotionless” appearance even in situations that would typically provoke an emotional response.	[33]	Affect_measure
Facial expression	“Sad” facial expressions, such as less frequent smiling, have been associated with the severity of depression.	[31,40,61-63]	Mouth_curvature and eyebrow_droop
Reduced eye contact	People with depression might avoid making eye contact, which can be a sign of feelings of worthlessness or guilt.	[29-32]	Gaze_down_dist, gaze_x_dist, and gaze_y_dist
Slow movements	Psychomotor retardation can manifest as slowed facial movements or reactions in those with depression.	[61]	Movement_speech_measure
Audio features			
Monotone speech	Individuals with depression might exhibit a lack of variability in pitch, leading to speech that sounds monotonous.	[18-22,27]	Pitch and pitch_std
Reduced speech volume	Speaking more softly or with less projection than usual can be indicative of depression.	[18,22,64-67]	Vol, vol_std, and ealvi
Reduced vocal prosody	A decrease in the rhythmic and melodic aspect of speech can be indicative of depression.	[18-20,24,25,28,64,68]	Jitter, shimmer, timbre, and formant
Long pauses	Depression might result in more increased pauses between words or sentences, reflecting hesitancy, slowed thinking, or difficulty in organizing thoughts.	[18,21,68,69]	Audio_pauses
Transcript features			
Long pauses	Depression might result in more increased pauses between words or sentences, reflecting hesitancy, slowed thinking, or difficulty in organizing thoughts.	[18,21,68,69]	Transcript_pauses
Slow speech rate	People with depression might speak more slowly, potentially reflecting slowed cognitive processing.	[22,23,68-71]	Words_per_s
Reduced responsiveness	A person with depression might be less verbally responsive in conversations, potentially taking longer to reply or offering shorter answers.	[21,22,69,70]	Response_latency and transcript_len
Decreased complexity and length of speech	Speech in individuals with depression might be less complex in terms of vocabulary and sentence structure, and individuals might be less talkative overall.	[28,34-37,39,40,67]	Complexity, emotion_keyword_prop, and sentiment_score

**Table 2.** Mapping features for fatigue.

Feature	Theoretical reasoning	References	Implemented features
Visual features			
Droopy eyelids	The muscles around the eyes may begin to sag due to tiredness, causing the eyelids to droop.	[72]	Eyelid_droop
Decreased blink rate	Fatigue can lead to a reduced rate of blinking.	[73-77]	Blinks_per_s and blink_len
Yawning	While yawning is a natural behavior, frequent yawning can be an overt sign of fatigue or drowsiness.	[76,78]	Yawns_per_s and yawn_len
Reduced expressiveness	Fatigue might cause an individual to have fewer facial movements or expressions.	[72]	Mouth_curvature
Audio features			
Decreased volume	A tired individual might speak more softly or with less energy than when they are well rested.	[8,79]	Vol and vol_std
Decreased pitch variability	A fatigued voice might sound more monotonous, with less variation in pitch.	[23,25]	Pitch and pitch_std
Flatter voice profile	Decreased variation and width of the voice’s spectral profile can be indicative of fatigue.	[25,79]	Timbre and formant
Increased pauses	There might be more frequent and longer pauses between words or sentences, reflecting slowed cognitive processing or the need to gather thoughts.	[23,79,80]	Audio_pauses
Transcript features			
Increased pauses	There might be more frequent and longer pauses between words or sentences, reflecting slowed cognitive processing or the need to gather thoughts.	[23,79,80]	Transcript_pauses
Slow speech rate	The overall rate of speech might decrease when a person is tired.	[23,80]	Words_per_s
Shortened responses	Fatigued individuals might offer shorter answers or engage less in conversation.	[23]	Transcript_len
Impaired memory recall	Fatigued individuals might struggle to remember certain words, names, or details, leading to more frequent use of filler words such as “um” or “uh.”	[80]	Hesitations_per_s

**Table 3.** Mapping features for attention and cognition.

Feature	Theoretical reasoning	References	Implemented features
Visual features			
Decreased blink rate	Blinking might increase when in periods of high attention demand during cognitive tasks.	[81]	Blinks_per_s
Eye movements	Eye movements outside the line of focus might be reduced when in a period of visual attention.	[82]	Eye_movements_per_s
Audio features			
Volume	Speech volume can indicate differences in arousal and fatigue.	[26]	Vol, vol_std, vol_range, and volume_slope
Pitch and pitch variability	Pitch might be less varied during periods of low engagement or emotional expressivity.	[26,83]	Pitch and pitch_std
Voice timbre	Voice might be breathier or more strained when lacking attention.	[26]	Jitter, shimmer, and timbre
Transcript features			
Reduced responsiveness	Response latency has been shown to enable the detection of the Stroop effect (delay in reaction time).	[84]	Response_latency

The webcam capture rate varied depending on a number of factors, including the user’s device and browser. As this capture rate was not controllable by the Okaya platform, the analysis had to handle landmark frames captured at nonregular times. Generally, the capture rate was 30 to 60 frames per second, but they were usually not captured at a consistent rate. All sessions were interpolated to a capture rate of 30 frames per second, where each time step was a linear interpolation between the 2 neighboring frames. Features were extracted in Python (Python Software Foundation) using the libraries *SciPy* and *NumPy*.

Similarly, audio capture rate varied per user, so all audio recordings were resampled to a sampling frequency of 22,050 Hz. Features were extracted in Python using the libraries *Librosa*, *NumPy*, *SciPy*, *Parselmouth*, and *Statsmodels*.

Text features were extracted in Python using the libraries *NumPy*, *Textacy*, and *Natural Language Toolkit* (Team NLTK). The exact implementation of each feature extractor is omitted as the details of the Okaya platform are proprietary.

Several temporal aggregations were used to calculate feature values: averaged per second, per frame, per response, or per detected event. The exact aggregation and unit used for each feature are listed in Multimedia Appendix 1.

## Results

### Engagement and Usability Results

Three participants provided qualitative and survey feedback following completion of their Okaya sessions. Full survey responses can be found in Multimedia Appendix 2. Overall, preliminary user satisfaction with the conversational platform was given a rating of between 4 and 5 out of 5 on overall experience and usability among all participants who provided feedback (3/3). These participants described the platform as intuitive and easy to navigate and reported feeling adequately supported during setup and use. One participant highlighted the responsiveness of the support team and appreciated having direct access to human assistance when needed.

Motivations for engagement included curiosity about the technology and the opportunity for structured self-reflection during check-ins. One participant noted that the conversations prompted personal insight and self-awareness, describing the process as a valuable moment to “intentionally reflect” on life aspects discussed during the session. Common barriers to more frequent engagement included time constraints, insufficient or poorly timed reminders, and occasional concerns about privacy when speaking in shared environments. Notably, all participants who completed the survey expressed overall confidence in data privacy and platform security, each giving this aspect a rating of 5 out of 5.

Suggestions for improvement focused on enhancing convenience and personalization, including text-based reminders with direct log-in links, greater mobile accessibility, and shorter baseline tasks prior to the conversational check-in. Survey participants also expressed interest in receiving personalized summaries or progress visualizations of their responses. All the participants who provided feedback indicated interest in participating in future sessions using the Okaya platform, reinforcing the platform’s acceptability and potential for longitudinal engagement within operational populations.

### Correlation Analysis

#### Overview

**Figure 3.**
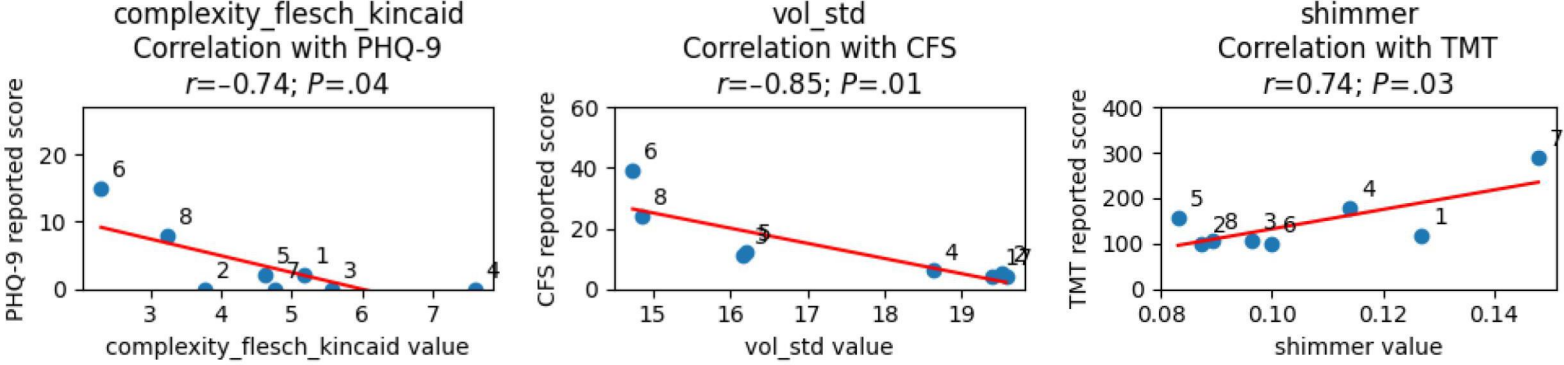
Most significant features for each measure and their associated correlation. CFS: Cancer Fatigue Scale; PHQ-9: Patient Health Questionnaire–9; TMT: trail making test.

Pearson correlations and their associated significance are listed below. Although every participant was included in the dataset and most completed multiple sessions, we only took into account the earliest sample for each participant for the correlation analysis. The earliest sample was used to illustrate the use of an initial sample as a baseline, upon which personalized models can be built, illustrated in the case study below. We also observed low intraparticipant variability for each clinical measure, which hindered the application of more sophisticated statistical models at this stage. For each feature, outliers (defined as any measurement more than 2 SDs from the mean) were removed. The most significant features are plotted against the associated measures in Figure 3.

#### Correlations for Depression

Exploratory correlation analyses revealed several significant associations between multimodal features and depressive symptom severity as measured using the PHQ-9. Acoustic and linguistic indicators showed the strongest relationships. In this small sample specifically, pitch (*r*=0.71; *P*=.047) and volume slope (*r*=0.78; *P*=.04) were positively correlated with PHQ-9 scores. This suggests preliminary patterns and warrants further exploration into predictive modeling to validate that greater variability and upward trajectories in vocal intensity may correspond to higher self-reported depression levels. In contrast, volume SD was negatively correlated (*r*=−0.73; *P*=.04), encouraging a possible exploration into how participants with greater depressive symptoms may exhibit reduced dynamic range in speech.

Textual complexity metrics—including the automated readability index (*r*=−0.71; *P*=.047), Flesch-Kincaid complexity (*r*=−0.74; *P*=.04), and Gunning Fog index (*r*=−0.72; *P*=.04)—also demonstrated negative correlations with PHQ-9 scores; thus, the individuals in our preliminary dataset reporting higher depressive symptoms tended to produce shorter or linguistically simpler utterances during conversational interactions. Due to the low sample size, it is impossible to say whether there are any actual meaningful correlations; however, it does warrant further exploration with larger sample sizes and more resilient analyses. Due to our limited sample size, individual correlations are also not expected to be reliable or generalizable at this stage. Together, these relationships support the feasibility of deriving speech- and language-based digital biomarkers of depression from naturalistic conversational data.

#### Correlations for Fatigue

Analysis of correlations between multimodal features and fatigue as measured using the CFS identified several significant acoustic, visual, and linguistic markers. Among acoustic features, pitch (*r*=0.84; *P*=.009), volume slope (*r*=0.84; *P*=.02), and average F2 formant frequency (*r*=0.76; *P*=.03) were positively correlated with fatigue severity, whereas volume SD (*r*=−0.85; *P*=.007) showed a strong negative association. This warrants further investigation into how individuals reporting higher fatigue might exhibit flatter, less dynamically modulated vocal patterns, consistent with decreased speech energy and prosodic variation observed in fatigue-related speech studies.

Additionally, the linguistic Gunning Fog index was inversely correlated with CFS scores (*r*=−0.71; *P*=.049), indicating that higher fatigue levels were associated with simpler or less complex speech production. One visual feature, eyelid droop (*r*=0.71; *P*=.047), also demonstrated a significant positive association with fatigue, consistent with prior literature linking ocular and facial muscle changes to tiredness. Together, these findings highlight the sensitivity of both speech acoustics and facial metrics to self-reported fatigue, supporting their potential use as digital biomarkers for real-time fatigue monitoring. However, due to the limited sample size and analysis, individual associations should not be interpreted as predictive or applicable to general populations at this stage.

#### Correlations for Attention and Cognition

Exploratory analyses examining associations between multimodal features and cognitive performance as measured using the TMT identified a single significant relationship. The acoustic feature shimmer, which captures cycle-to-cycle variability in vocal amplitude, was positively correlated with TMT scores (*r*=0.74; *P*=.03). This association points to greater irregularity in voice amplitude when participants had longer task completion times. Further research into how lower cognitive efficiency might potentially relate to fatigue-related motor or attentional variability in speech production may be worthwhile. Although preliminary, this finding aligns with those of prior literature linking changes in vocal stability to fluctuations in attention and executive functioning. These results indicate that fine-grained acoustic features derived from conversational speech may offer a feasible, low-burden proxy for cognitive performance monitoring in remote or operational settings. Once again, although the exact associations found should not be treated as reliable or generalizable at this stage, this does suggest a potential future study with larger samples and analyses.

### Descriptive Summary of Significant Associations

To complement the quantitative analyses, we conducted a descriptive review of the distribution of significant multimodal features across clinical domains. As shown in Table 4, depression-related features were primarily linguistic and acoustic in nature, with no significant visual markers identified. Fatigue exhibited the broadest feature coverage, encompassing 1 visual variable, 4 acoustic variables, and 1 linguistic variable. When examining fatigue subscales, distinct modality patterns emerged: the physical and affective fatigue components were each associated with multiple acoustic features, whereas the cognitive subscale was linked to both visual and acoustic indicators. In contrast, cognitive performance as measured using the TMT was primarily associated with a single acoustic feature, reflecting a narrower signal profile. It is important to note that feature set size also had an effect; for example, fatigue measures displayed a clear feature representation across audio features, but audio features also made up a larger proportion of the total features compared to visual and text features. There is no clear evidence of any specific feature modality showing broader significance or sensitivity.

**Table 4.** Count of significant features for each subscale of the clinical measures and feature type out of the total number of features tested.

Clinical measure and subscale	Feature categories, n (%)		
	Visual features (n=13)	Audio features (n=28)	Text features (n=25)
PHQ-9^a^	0 (0.0)	3 (10.7)	3 (12.0)
CFS^b^	1 (7.7)	4 (14.3)	1 (4.0)
Physical	1 (7.7)	4 (14.3)	3 (12.0)
Affective	0 (0.0)	5 (17.9)	0 (0.0)
Cognitive	1 (7.7)	5 (17.9)	0 (0.0)
TMT^c^	0 (0.0)	1 (3.6)	0 (0.0)
Part A	0 (0.0)	0 (0.0)	1 (4.0)
Part B	0 (0.0)	1 (3.6)	0 (0.0)

a PHQ-9: Patient Health Questionnaire–9.

b CFS: Cancer Fatigue Scale.

c TMT: trail making test.

### Risk Score Case Study

To explore the feasibility of individualized monitoring, we conducted case studies on participants who completed multiple Okaya sessions. For each individual, a composite “risk score” was calculated by standardizing the significant multimodal features (*z*-scoring within participants) and summing the deviations from the mean, with directionality determined by the feature’s correlation with the clinical measure. This approach provided a demonstration of an interpretable, participant-specific index of deviation from baseline functioning across time; however, due to the low sample size and unstable correlations, it is purely used as a demonstration in this case, not a proof of actual meaningful capability.

An example use case can use a longitudinal visualization of these risk scores to reveal dynamic fluctuations in both depression- and fatigue-related indexes across sessions. For example, in participants with higher variability, increases in risk score can correspond to periods of increased self-reported symptom severity, whereas lower or stable values can align with more consistent well-being reports. Although exploratory, these case studies illustrate how multimodal conversational data can support within-person anomaly detection and temporal tracking of psychological and physiological states. This example provides a demonstration of how individualized, feature-based composite scores may offer a viable framework for early identification of changes in mood, fatigue, or cognitive function in remote monitoring contexts.

**Figure 4.**
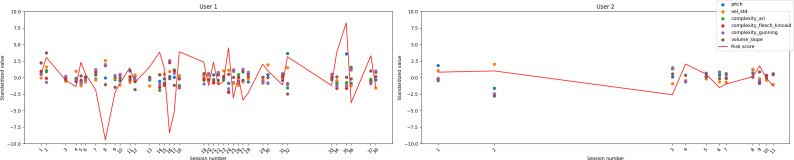
Risk score case study for depression.

Data on depression and fatigue for the 2 participants with the most sessions are shown in Figures4  5, respectively (TMT only exhibited 1 significant feature and, as such, a composite score was not meaningful).

**Figure 5.**
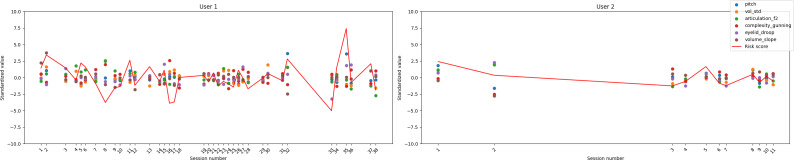
Risk score case study for fatigue.

## Discussion

### Principal Findings

We found several multimodal features that correlated with depression, fatigue, and cognition, some of which extended beyond patterns reported in prior literature. As expected, depression was characterized by reduced variability in vocal intensity and lower linguistic complexity, consistent with psychomotor slowing and affective flattening. Fatigue demonstrated a broader multimodal signature involving both acoustic and visual domains, including reduced volume variability, higher pitch, and greater eyelid droop, reflecting decreased energy and arousal. Notably, volume slope, a feature we did not find in the literature, was significant for both depression and fatigue, suggesting that individuals with depression or fatigue might have noticeable patterns in how speech volume progresses over time, especially during utterances provided on the Okaya platform. Additionally, complexity, a feature previously linked to depressive speech, was found to correlate with fatigue, indicating that depression and fatigue may share some similar effects on word choice and diversity [28,36]. Together, these findings highlight the potential for future study of established digital biomarkers in the Okaya platform and the emergence of cross-domain signals that may bridge affective, cognitive, and fatigue-related processes.

The findings of this feasibility study indicate that the Okaya conversational platform is both usable and acceptable for collecting multimodal data in an operational setting. Participants demonstrated relatively sustained engagement, with most completing multiple sessions, suggesting that the platform’s conversational format and remote accessibility supported ongoing participation. Usability feedback further underscored this positive experience—participants rated the system highly in terms of ease of use, clarity of instructions, and overall satisfaction. Qualitative feedback revealed that users found the interaction intuitive and meaningful, often describing the check-in as an opportunity for reflection rather than a burdensome task. Importantly, participants expressed high confidence in data privacy and security, which is critical for user trust in AI-enabled health technologies [85-87]. At the same time, feedback highlighted opportunities for improvement, including more flexible scheduling options, reminders, and greater mobile accessibility. Together, these findings suggest that Okaya’s design successfully balanced data collection rigor with user comfort and autonomy, supporting its feasibility for repeated deployment in diverse and high-performance populations. These results provide an encouraging foundation for the platform’s next phase of validation and refinement, emphasizing scalability, personalization, and long-term adherence.

The findings from the case study align closely with the long-term vision for the Okaya platform. The observed fluctuations in individualized risk scores and their correspondence with self-reported symptom changes demonstrate the platform’s potential for longitudinal, within-person monitoring—a central principle behind the Okaya Index and the simplified risk score we presented. Rather than relying on evidence from population-based studies, the Okaya platform emphasizes personalized baselines and interprets deviations relative to each individual’s unique behavioral and physiological profile. This individualized approach reflects the platform’s goal of enabling early identification of shifts in emotional, cognitive, or fatigue states. As the platform evolves, integration of these multimodal risk scores into the Okaya Index will enable continuous refinement of trajectories across time and populations, ultimately supporting adaptive, AI-driven insights that can inform both clinical decision-making and support for operational populations ranging from pilots to firefighters. This trajectory aligns with the goal of advancing proactive mental health and performance optimization in real-world settings.

From a clinical and research perspective, these findings illustrate the promise of conversational, multimodal sensing as a foundation for precision mental health and digital phenotyping. By capturing subtle changes in voice, facial behavior, and language over time, systems such as Okaya can complement traditional self-report and clinician-administered tools, providing a richer, continuous view of mental and cognitive functioning. Such individualized, data-driven assessments have potential applications across health care, occupational performance, and behavioral health monitoring, particularly in cases in which real-time insight and early intervention are critical. Importantly, the Okaya platform’s emphasis on *nondiagnostic* and *privacy-preserving* analytics aligns with emerging standards for responsible AI in health technology [13,88,89]. As digital biomarkers move closer to clinical integration, continued validation across diverse populations and conditions will be essential to ensure generalizability, interpretability, and equity [85,86,90]. Ultimately, this study of the Okaya platform represents a step toward an ethically grounded, adaptive ecosystem for mental health monitoring—one that transforms multimodal data into actionable insights while safeguarding user trust and autonomy.

This study has several limitations that should be considered when interpreting the findings. First, the sample size was small and limited due to our focus on operational populations, which are harder to recruit, constraining generalizability and the ability to model interindividual variability. Thus, the correlations we report are not meant to be statistically resilient or generalizable at this stage. Therefore, these findings preclude reliable statistical inference of robust estimates of the correlations in these data and, rather, suggest future avenues for discovery. Second, although validated instruments such as the PHQ-9, CFS, and TMT were used for comparison, these measures may not fully capture the temporal dynamics or subtle within-person fluctuations that multimodal digital biomarkers are designed to detect. Traditional self-report and performance-based assessments tend to be episodic and relatively stable, which may underestimate moment-to-moment variability in affect, fatigue, and cognition. Future studies should incorporate higher-resolution clinical measures—such as ecological momentary assessment or brief daily self-reports—that provide richer temporal data and enable stronger coupling between behavioral features and self-reported states [1-3,87,91]. Integrating these temporally aligned measures would allow for more precise modeling of intraindividual change and enhance the ecological validity of the derived digital biomarkers. Finally, while the Okaya platform demonstrated feasibility and acceptability in this study, further validation with larger, more diverse samples and extended longitudinal follow-up is necessary for predictive modeling and design of personalized remote monitoring systems.

### Conclusions

The conversational and AI-enabled platform (Okaya) was feasible among an operational sample from the US Air and Space Force as a method for collecting multimodal data correlated with depression, fatigue, and cognition. Future work will examine larger samples with repeated measures to assess the test-retest reliability and predictive validity of multimodal digital biomarkers. Ultimately, the goal of the platform is the development of personalized models for each user while detecting anomalies in a remote monitoring setting.

## Supplementary material

10.2196/87054 Multimedia Appendix 1 Feature list.

10.2196/87054 Multimedia Appendix 2 Survey responses.

## Abbreviations

AI: artificial intelligence
CFS: Cancer Fatigue Scale
PHQ-9: Patient Health Questionnaire–9
TMT: trail making test
USAF: US Air Force
