# Supplementary material for: A Conversational Platform (Okaya) for Multimodal Digital Biomarkers of Fatigue, Cognition, and Mental Health: Feasibility Observational Study
Source: JMIR Form Res. 2026 Apr 1;10:e87054. doi: 10.2196/87054 (PMC13043011; doi:10.2196/87054)
Supplement: Multimedia Appendix 1 [file formative-v10-e87054-s001.docx]

## **Feature List**

| **Feature** | **Description** | **Unit** | **Aggregation** |
| --- | --- | --- | --- |
| *Visual Features* | | | |
| blinks_per_s | The number of detected blinks per second using a proprietary eye aspect ratio and peak detection algorithm | blinks/second | Average per-second |
| blink_len | Average blink duration | seconds | Average per-blink |
| eyelid_droop, eyebrow_droop | How much the eyelids and eyebrows droop | N/A (MediaPipe-specific) | Average per-frame |
| gaze_down_dist, gaze_x_dist, gaze_y_dist | How far the eyes gaze down or away from center | N/A (MediaPipe-specific) | Average per-frame |
| eye_movements_per_s | The number of detected sharp eye movements per second | movements/second | Frequency |
| yawns_per_s | The number of detected yawns per second using a proprietary mouth aspect ratio and peak detection algorithm | yawns/second | Average per-second |
| yawn_len | Average yawn duration | seconds | Average per-yawn |
| mouth_curvature | Average magnitude of smiling or frowning | N/A (MediaPipe-specific) | Average per-frame |
| movement_speed_measure | Derivative (speed) of MediaPipe blendshapes | N/A (MediaPipe-specific) | Average per-frame |
| affect_measure | Overall amount of facial expression | N/A (MediaPipe-specific) | Average per-frame |
| *Audio Features* | | | |
| pitch | Mean of the fundamental frequency the user spoke at | Hz | Average per-frame |
| pitch_std | Standard deviation of the fundamental frequency the user spoke at | Hz | Average per-frame |
| jitter | Variation in audio frequency | % | Average per-frame |
| shimmer | Variation in audio amplitude | % | Average per-frame |
| vol | Mean of speech volume | dB | Average per-frame |
| vol_std | Standard deviation of speech volume | dB | Average per-frame |
| vol_range | Difference between maximum and minimum speech volume | dB | Average per-frame |
| crest_factor | Ratio of peak volume to average volume | N/A | Average per-frame |
| volume_slope | The slope of the regression line between the beginning and end of an audio recording | dB/s | Average per-recording |
| ealvi | Emotional Arousal Level Voice Index, which describes the “roughness” of the frequency waveform. Increases as the proportion of acceleration to the average frequency increases. | N/A | Average per-frame |
| timbre_centroid, timbre_rolloff | Timbral measurements of the audio, describing the distribution of frequencies across the frequency decomposition, and thus the “color” or quality of tone in speech | Hz | Average per-frame |
| timbre_flatness, timbre_affinity, timbre_sharpness, timbre_spectral_skewness,timbre_spectral_kurtosis, timbre_spectral_entropy |  | N/A |  |
| timbre_harmonicity, timbre_spectral_tilt |  | dB |  |
| formant_f1, formant_f2, formant_f3, formant_f4, formant_f5 | Average F1, F2, F3, F4, and F5 formant frequencies | Hz | Average per-frame |
| audio_pauses_per_s | The number of pauses, detected from audio silences, per second | pauses/second | Average per-second |
| audio_pause_len | The average pause length, detected from audio silences | seconds | Average per-pause |
| audio_pause_len_std | Standard deviation of pause length, detected from audio silences | seconds | Average per-pause |
| *Transcript Features* | | | |
| response_latency | Average time it took to respond to Sanora | seconds | Average per-response |
| words_per_s | Number of words spoken per second | words/second | Average per-second |
| transcript_len | Total number of words spoken | words | Average per-response |
| hesitations_per_s | Number of hesitations (”uh,” ”um,” etc.) per second | hesitations/second | Average per-second |
| complexity_ttr, complexity_log_ttr, complexity_segmented_ttr, complexity_ari, complexity_coleman_liau, complexity_flesch_kincaid, complexity_gunning, complexity_smog | Various measurements for text complexity, including TTR, log TTR, segmented TTR, ARI, Coleman-Liau Index, Flesch-Kincaid Index, Gunning Index, and SMOG Index | Grade level (require to read and understand the text) | Average per-response |
| transcript_pauses_per_s | The number of pauses, detected from transcribed word timings, per second | pauses/second | Average per-second |
| transcript_pause_len | The average pause length, detected from transcribed word timings | seconds | Average per-pause |
| transcript_pause_len_std | Standard deviation of pause length, detected from transcribed word timings | seconds | Average per-pause |
| emotion | Proportion of words that are associated with a certain emotion, including anger, anticipation, disgust, fatigue, fear, joy, sadness, surprise, and trust | % | Average per-response |
| sentiment_score | Positive (+1) or negative (-1) sentiment, scored by the VADER algorithm | N/A | Average per-response |

### 
