# Supplementary material for: A Conversational Platform (Okaya) for Multimodal Digital Biomarkers of Fatigue, Cognition, and Mental Health: Feasibility Observational Study
Source: JMIR Form Res. 2026 Apr 1;10:e87054. doi: 10.2196/87054 (PMC13043011; doi:10.2196/87054)
Supplement: Multimedia Appendix 2 [file formative-v10-e87054-s002.docx]

## **Survey Responses**

| **Question** | **Participant 1 Answer** | **Participant 2 Answer** | **Participant 3 Answer** |
| --- | --- | --- | --- |
| Overall Experience: How would you rate your overall experience using the Okaya platform during the trial? (1-5) | 5 | 5 | 4 |
| Usability: How easy was it to navigate and use the Okaya platform? (1-5) | 5 | 5 | 5 |
| Engagement: What motivated you to complete check-ins? | I appreciate having the ability to intentionally reflect on things I was asked about during a check in. It made me realize that there was some room for improvement in my life. | Curiosity | New tech never seen this way of mental health assessment so I am hopeful |
| Engagement: What barriers prevented you from doing so more frequently? | Privacy concerns, I wasn't reminded enough, or I was reminded at the wrong time, The privacy concerns weren’t about my data being protected. It was having a private place to speak to Senora that was a barrier a few times. Not a huge deal. | Time constraints | I didn't feel like I needed to check in, I wasn't reminded enough, or I was reminded at the wrong time |
| If you selected one of the above options, please expand on your experience or what could be done to help you overcome those barriers. | A reminder text with a link to my login screen would’ve been helpful. Most times I just forget to do a check in. | Job issues | More frequent reminders. The first part where you have to connect numbers and letters was too long. |
| Privacy and Security: How confident are you in the privacy and security measures of the Okaya platform? (1-5) | 5 | 5 | 5 |
| Privacy and Security: Were there any concerns regarding data privacy or security that you encountered during the trial? | Not at all | None. I liked the lock code for the answers and the discussion. | A little. I was mindful of what I said because I was not comfortable opening up too much to an A.I |
| Support and Resources: How would you rate the support and resources provided by the Okaya team during the trial? (1-5) | 5 | 5 | 5 |
| Support and Resources: Did you feel adequately supported in understanding and using the platform? If not, what additional support would have been helpful? | Yes. I really appreciated how easy it was to get ahold of a human being to help me directly. | Excellent support ! | Yes |
| Suggestions for Improvement: What features or improvements would you like to see in future versions of the Okaya platform? Any additional comments or suggestions for the Okaya team? | I’d love a reminder text. Additionally, I really liked that I could have a summary generated of my mental health journey. Highlighting the things I could improve on will be invaluable in my future. | More interaction. I liked the answers, but I thought they could have presented more options wrt suggestions to dealing with problems. | Reduce the amount of puzzles you have to do to baseline yourself. Make the website more appealing friendly, most younger people are familiar with doing things on smart phones vs laptops. |
| Future Involvement: Would you be interested in participating in another trial this year? | Yes | Yes | Yes |
